# Elevated Intraocular Pressure after Descemet Stripping Automated Endothelial Keratoplasty in Patients with a Trabeculectomy: A Case Series

**DOI:** 10.5005/jp-journals-10008-1193

**Published:** 2016-02-02

**Authors:** Mariana Patricia Hardin Sheales, Elsie Chan, Ghee Soon Ang, Yu Xiang George Kong

**Affiliations:** Registrar, Department of Ophthalmology, The Royal Victorian Eye and Ear Hospital, Melbourne, Australia; Consultant, Department of Ophthalmology, Corneal Unit, The Royal Victorian Eye and Ear Hospital; Centre for Eye Research Australia, University of Melbourne, Melbourne, Australia; Consultant, Department of Ophthalmology, Glaucoma Investigation Research Unit, The Royal Victorian Eye and Ear Hospital Melbourne, Australia; Glaucoma Fellow, Department of Ophthalmology, Glaucoma Investigation Research Unit, The Royal Victorian Eye and Ear Hospital Melbourne, Australia

**Keywords:** Cornea, Descemet stripping automated endothelial keratoplasty, Glaucoma, Intraocular pressure, Trabeculectomy.

## Abstract

We report a case series of three patients with previous trabeculectomies who developed elevated intraocular pressure (IOP) in the immediate postoperative period after routine Descemet stripping automated endothelial keratoplasty (DSAEK). All patients had functioning trabeculectomies preoperatively, and developed elevated IOP between 41 and 69 mm Hg within 24 hours following DSAEK surgery. The IOP was successfully controlled in all patients with topical IOP-lowering medications and oral acetazolamide, with the addition of ocular massage and release of aqueous for two patients. Thereafter, all patients maintained well-controlled IOPs. Patients with trabeculectomies should be followed-up closely immediately after DSAEK to monitor for raised IOP. The mechanism for this pressure rise is uncertain, but may involve air in the trabeculectomy sclerostomy or bleb resulting in blockage of aqueous flow.

**How to cite this article:** Sheales MPH, Chan E, Ang GS, Kong YXG. Elevated Intraocular Pressure after Descemet Stripping Automated Endothelial Keratoplasty in Patients with a Trabeculectomy: A Case Series. J Curr Glaucoma Pract 2015;9(3):100-103.

## INTRODUCTION

Corneal decompensation in patients with a history of glaucoma and filtering trabeculectomies may necessitate descemet stripping automated endothelial keratoplasty (DSAEK) surgery. At the end of the procedure, air is routinely used as a tamponade to facilitate adhesion of the endothelial graft to the host’s corneal stroma. A few reports have noted air in the trabeculectomy bleb postoperatively, resulting in hypotony and subsequent graft dislocation.^1-3^ Marked elevation of intraocular pressure (IOP) immediately post-DSAEK in the setting of a functioning filtration bleb has not been described.

## METHODS

### Surgical Technique

Surgeries, in the cases described in this series, were performed under peribulbar block (case 1) or general anesthesia (cases 2 and 3). Descemet’s membrane was scored and stripped under balanced salt solution (BSS). The donor lenticule was inserted using a Busin glide (Moria SA, Antony, France) and a complete (100%) air fill maintained for 10 minutes. Residual fluid from the graft-host interface was expressed through venting incisions in each of the four quadrants prior to air-BSS exchange. All wounds were sutured with 10/0 Nylon or Polyglactin 910 (Vicryl®, Ethicon, Somerville, New Jersey, United States of America). Following surgery, the patients were instructed to remain supine for a further 2 hours. Patients were prescribed phenylephrine 0.12%/prednisolone acetate 1% (Prednefrin Forte®, Allergan, Australia Pty Ltd., Gordon, New South Wales, Australia) every 2 hours and chloramphenicol 0.5% (Chlorsig®, Aspen Pharma Pty Ltd, St Leonards, New South Wales, Australia) four times a day. Deviations from this surgical technique are included in the case descriptions below:

## CASE REPORTS

The key features of these cases are summarized in Table 1.

### Case 1

A 74-year-old female had a history of hypermetropia (axial length of 21.0 mm), laser peripheral iridotomy and previous cataract surgery complicated by aqueous misdirection in the left eye. The aqueous misdirection was treated at the time with a laser peripheral iridotomy, capsulotomy and disruption of the anterior hyaloid face, followed by an anterior vitrectomy and trabeculectomy augmented with mitomycin C. She subsequently developed pseudophakic bullous keratopathy (PBK) and underwent a DSAEK 3 years later. The preoperative IOP was 15 mm Hg, with a visual acuity of ‘hand movements ’. Due to a shallow anterior chamber at the beginning of surgery, intravenous mannitol (200 ml 20% in 1L of compound sodium lactate) was administered. An anterior vitrector was then used to remove Elschnig’s pearls and to create an inferior peripheral iridotomy. The case otherwise proceeded as described above. At the end of the case, a 100% air fill was maintained. When the patient was reviewed 1.5 hours following surgery, she was complaining of ocular pain. The IOP was 35 mmHg, the graft was attached with a 50% air fill, and the anterior chamber was deep. Her IOP was rechecked 1 hour later and found to be 69 mm Hg, at which time she was given 500 mg oral acetazolamide (Diamox®, Aspen Pharma Pty Ltd, St Leonards New South Wales, Australia), topical brimonidine tartrate 0.2%/timolol 0.5% (Combigan®, Allergan Australia Pty Ltd, Gordon New South Wales, Australia) and latanoprost 0.005%/timolol 0.5% (Xalacom®, Pfizer Australia Pty Ltd, West Ryde New South Wales, Australia). Ninety minutes later, the IOP was 45 mm Hg so ocular massage was performed and aqueous was released from the side port, which decreased the IOP to 20 mm Hg (5.6 hours postoperatively). The next day, the IOP was 12 mm Hg and has remained below 20 mm Hg over the postoperative period. Oral acetazolamide was continued 250 mg BD for 2 more days and topical brimonidine tartrate 0.2%/timolol 0.5% BD for 4 days. At her most recent follow-up visit 8 weeks following surgery, the IOP was 16 mm Hg without any IOP-lowering medications, the graft has remained attached with no other complications recorded. The uncorrected visual acuity (UCVA) was 6/30.

**Table 1:** **Table 1:** Summary of key features of three cases of patients with trabeculectomies and elevated IOP post-DSAEK

*Case*		*1*		*2*		*3*	
Age		74		88		74	
Gender		Female		Male		Female	
Laterality (eye)		Left		Right		Right	
Glaucoma type		Aqueous misdirection		Pseudoexfoliation		Pseudoexfoliation	
Indication for DSAEK		PBK		PBK		PBK	
Preoperative IOP		15 mm Hg*		5 mm Hg*		8 mm Hg*	
Preoperative VA		HM		CF		HM	
Preoperative ophthalmic medications		Phenylephrine 0.12%/ prednisolone acetate 1% daily		Nil		Fluorometholone acetate 0.1% TDS Vancomycin 5% QID Paraffin TDS	
Trabeculectomy bleb morphology		Diffuse		Diffuse		Flat	
Number of years post-trabeculectomy		3		10		9	
Maximal postoperative IOP/number of hours postoperative		69 mm Hg*/2.5 hours		45 mm Hg*/4.2 hours		41 mm Hg*/16.8 hours	
Postoperative treatment		Brimonidine tartrate 0.2%/timolol 0.5% BD		Latanoprost 0.005% daily		Brimonidine tartrate 0.2%/timolol 0.5% stat	
		Latanoprost 0.005%/timolol 0.5% nocte		Apraclonidine 0.5% TDS		Latanoprost 0.005%/ timolol 0.5% stat	
		Acetazolamide 500 mg PO stat		Acetazolamide 250 mg PO QID		Acetazolamide 250 mg PO stat	
		Ocular massage		Ocular massage			
		Release of aqueous		Release of aqueous			
First IOP reading < 21 mm Hg^‡^/number of hours post-treatment initiation		20 mm Hg^†^/5.6 hours		20 mm Hg*/6.0 hours		14 mm Hg*/4.5 hours	
Ongoing postoperative IOP medications		Nil		Nil		Brimonidine tartrate 0.2%/timolol 0.5% BD Latanoprost 0.005% nocte	
IOP at last review/number of weeks Post-DSAEK		16 mm Hg^†^/8 weeks		7 mm Hg*/6 weeks		16 mm Hg*/9 weeks	
VA at last review		6/30, PH 6/18		6/24, PH 6/15		6/36, PH no improvement	

Cmovements; IOP: Intraocular pressure; PBK: Pseudophakic bullous keratopathy; PH: Pinhole; VA: Visual acuity; *Intraocular pressure measured by rebound tonometry Icare® (Icare Finland Oy, Vantaa, Finland); ^†^Intraocular pressure measured by Goldmann applanation tonometry; ^‡^Normal intraocular pressure < 21 mm Hg

### Case 2

An 88-year-old male had a history of pseudoexfoliation glaucoma, laser peripheral iridotomy, cataract surgery and a trabeculectomy (with two revisions, both with mitomycin C) in the right eye. Descemet stripping automated endothelial keratoplasty was performed for PBK 10 years after the most recent glaucoma operation. The preoperative IOP was 5 mm Hg and visual acuity was ‘counting fingers ’. At the end of surgery, an 80% air bubble was left in the anterior chamber. 4.2 hours postoperatively the patient developed ocular pain and was reviewed, the IOP was 45 mm Hg, the graft was attached with 50% air fill, and the anterior chamber was deep. Aqueous was released through a side-port incision and the pressure reduced to 20 mm Hg. 12 hours later, the pressure was once again elevated at 38 mm Hg so the patient was started on oral acetazolamide 250 mg QID, topical latanoprost 0.005% (Xalatan®, Pfizer Australia, West Ryde, New South Wales, Australia) daily and apraclonidine 0.5% (Iopidine®, Alcon Laboratories Pty Ltd, Frenchs Forest, New South Wales, Australia) TDS, and postured supine for 15 minutes. As the IOP remained elevated, bleb massage was performed, resulting in an IOP of 19 mm Hg. The IOP was 10 mm Hg 1.5 days postoperatively and has remained well controlled since IOP-lowering medications were ceased 8 days later. The most recent IOP 6 weeks post-DSAEK was 7 mm Hg with an UCVA 6/24, with no other graft-related complications.

### Case 3

A 74-year-old female had a history of cataract surgery and a trabeculectomy augmented with mitomycin C for pseudoexfoliation glaucoma in the right eye. She developed PBK and was scheduled for DSAEK 9 years after the original trabeculectomy. Unfortunately, she developed Staphylococcus epidermidis keratitis while awaiting surgery and was treated with topical vancomycin 5% QID (compounded at the authors hospital) and fluorometholone acetate 0.1% (Flarex® Alcon Laboratories Pty Ltd, Frenchs Forest, New South Wales, Australia) TDS. Preoperatively, her IOP was 8 mm Hg and visual acuity was ‘hand movements ’. At the end of the procedure the anterior chamber contained an 80% air bubble. Her IOP 2.2 hours postoperatively was 32 mm Hg, the graft was attached with 40% air fill, and the anterior chamber was deep. The next morning her IOP was 41 mm Hg, and, at this point, she was treated medically with oral acetazolamide 250 mg and brimonidine tartrate 0.2%/timolol 0.5%, and latanoprost 0.005%/timolol 0.5%. 4.5 hours later her IOP came down to 14 mm Hg. Postoperatively, she has remained on brimonidine tartrate 0.2%/timolol 0.5% BD and latanoprost 0.005% nocte due to her advanced glaucoma (cup-to-disk-radio 0.95 with advanced visual field loss). At her most recent follow-up visit 9 weeks postoperatively, her UCVA was 6/36 and IOP was 16 mm Hg. No other graft-related complications have been noted.

## DISCUSSION

Endothelial decompensation is an uncommon but visually significant long-term sequelae of glaucoma filtration surgery.^4^ We report three cases of significant IOP elevation in patients with functioning trabeculectomies occurring within 24 hours following routine DSAEK surgery for endothelial decompensation. This has not been reported in the literature despite a large case series with 113 patients undergoing Descemet’s stripping endothelial keratoplasty (DSEK) with previous glaucoma surgery (37 with previous trabeculectomy, 61 with previous tube shunt and 15 with both).^5^

We postulate the mechanism of raised IOP may be due to air injected at the time of surgery filtering into the bleb and, therefore, blocking the filtration channels of the trabeculectomy, including the sclerostomy. In all cases, the air bubble was significantly smaller postoperatively compared to the size of the air bubble that remained in the anterior chamber at the conclusion of surgery, greater than expected from absorption alone. It has previously been reported in the literature that air can filter out through the trabeculectomy, causing hypotony.^1-3^ It is possible that our patients may have had narrower filtration channels such that the surface tension of the air prevents the passage of air bubbles, causing obstruction to aqueous drainage. In patients with very limited alternative aqueous drainage facility, this would lead to marked IOP rise. Of note, none of our patients developed pupillary block, and preoperatively all patients had good IOP control without medications, suggesting that they had functioning trabeculectomies prior to DSAEK.

Although the impact of transient acute postoperative IOP spikes in glaucoma patients has not been clearly defined, the likelihood of causing additional permanent glaucomatous optic nerve damage remains. To reduce the risk of acute IOP elevation post-DSAEK, a reduced volume of air could be used for postoperative tamponade, but this may lead to a higher graft dislocation rate.^3,6^ Another possibility is for the patient to adopt the Trendelenburg position following DSAEK. The Trendelenburg position has been reported to prevent graft dislocation in the context of air escaping through the filtering bleb. Belkin et al successfully used this technique in a patient with repeated graft dislocations.^1^ This position moves the air inferiorly in the anterior chamber, away from the trabeculectomy site while still maintaining adequate tamponade.

This small case series highlights the importance of closely monitoring patients with trabeculectomies who undergo DSAEK as they may develop both high and low IOPs in the immediate postoperative period.

## References

[1]  Belkin A, Nesher R, Segev F (2013). Postoperative trendelenburg position for graft adherence after Descemets stripping automated endothelial keratoplasty.. Cornea.

[2]  Goshe J, Terry M, Li J, Straiko M, Davis-Boozer D (2012). Graft dislocation and hypotony after Descemet’s stripping automated endothelial keratoplasty in patients with previous glaucoma surgery.. Ophthalmology.

[3]  Ide T, Yoo S, Leng T, O ’Brien T (2009). Subconjunctival air leakage after Descemet’s stripping automated endothelial keratoplasty in a post-trabeculectomy eye.. Open J Ophthalmol.

[4]  Hau S, Barton K (2009). Corneal complications of glaucoma surgery.. Curr Opin Ophthalmol.

[5]  Aldave A, Chen J, Zaman A, Deng S, Yu F (2014). Outcomes after DSEK in 101 eyes with previous trabeculectomy and tube shunt implantation.. Cornea.

[6]  Esquenazi S, Rand W (2010). Safety of DSAEK in patients with previous glaucoma filtering surgery.. J Glaucoma.

